# Production and stability of cultured red blood cells depends on the concentration of cholesterol in culture medium

**DOI:** 10.1038/s41598-024-66440-z

**Published:** 2024-07-06

**Authors:** M. J. A. G. Claessen, N. Yagci, K. Fu, E. Brandsma, M. J. Kersten, M. von Lindern, E. van den Akker

**Affiliations:** 1 Department Research, Sanquin Blood Supply, Plesmanlaan 125, 1066 CX Amsterdam, The Netherlands; 2 Department of Hematology, Amsterdam University Medical Centers, Cancer Center Amsterdam, De Boelelaan 1117, 1081HV Amsterdam, The Netherlands; 3 Landsteiner Laboratory, Amsterdam University Medical Center, Plesmanlaan 125, 1066 CX Amsterdam, The Netherlands; 4 Present Address: Department of Life Sciences, Saxion University of Applied Sciences, M.H. Tromplaan 28, 7513AB Enschede, The Netherlands

**Keywords:** Cell biology, Molecular biology

## Abstract

The production of cultured red blood cells (cRBC) for transfusion purposes requires large scale cultures and downstream processes to purify enucleated cRBC. The membrane composition, and cholesterol content in particular, are important during proliferation of (pro)erythroblasts and for cRBC quality. Therefore, we tested the requirement for cholesterol in the culture medium during expansion and differentiation of erythroid cultures with respect to proliferation, enucleation and purification by filtration. The low cholesterol level (22 µg/dl) in serum free medium was sufficient to expand (pro)erythroblast cultures. Addition of 2.0 or 5.0 mg/dL of free cholesterol at the start of differentiation induction inhibited enucleation compared to the default condition containing 3.3 mg/dl total cholesterol derived from the addition of Omniplasma to serum free medium. Addition of 5.0 mg/dl cholesterol at day 5 of differentiation did not affect the enucleation process but significantly increased recovery of enucleated cRBC following filtration over leukodepletion filters. The addition of cholesterol at day 5 increased the osmotic resistance of cRBC. In conclusion, cholesterol supplementation after the onset of enucleation improved the robustness of cRBC and increased the yield of enucleated cRBC in the purification process.

## Introduction

Although generally safe, blood transfusion medicine faces a few major challenges^1^. Besides the ABO^2^ and Rh^3^ blood group systems, 45 blood group systems are known, which encode 362 antigens^4^. Alloimmunization is a serious risk for patients requiring recurrent transfusions, especially for patients with rare blood groups^5^. The generation of ex vivo cultured red blood cells (cRBCs) for transfusion purposes holds great promise to overcome such challenges in future blood transfusion medicine^6–12^.

The production of cRBC for transfusion purposes requires large scale expansion of progenitors and their maturation to hemoglobinized, enucleated reticulocytes that are stable and deformable to passage through the capillary beds in tissues. This implies the development of cost-effective media^13,14^ and bioreactor conditions^15,16^. It also requires the establishment of downstream bioprocessing, for instance to separate the extruded nuclei (pyrenocytes) from enucleated reticulocytes^13^. The latter process currently depends on leukocyte depletion filters that are already in use in the processing of blood donations to transfusion units^17^.

Stability and deformability of reticulocytes are important characteristics that affect the success of downstream processing of cRBC. The plasma membrane of RBC is linked to the erythroid specific submembraneous cytoskeleton of spectrin and ankyrin by the junctional and ankyrin/protein4.2 protein complex^18,19^. Approximately half of the mass of a RBC is represented by lipids (primarily phospholipids and non-esterified cholesterol) and its balanced composition confers resistance to shear stress in the circulation^20–23^.

Erythroblasts obtain their lipids either by biosynthesis or by absorption from various lipoprotein complexes^24^. Apart from triglyceride lipids that form the lipid bilayer, cholesterol is the most abundant lipid in the plasma membrane^25^. Deficiencies in the cholesterol biosynthesis are associated with anemia^26–29^.

During in vitro culture, the lipid composition and integrity of the cRBC plasma membrane depends on the presence of lipid carriers in the culture media. These may be lipoproteins present in serum or synthetic liposomes composed of bovine or human albumin, egg cholesterol and soybean lecithin^30,31^. Serum free culture media have to be supplemented by cholesterol to promote both expansion of the erythroblast compartment and differentiation to mature cRBC. Reported cholesterol supplementation in the media ranges from 0.02 up to 7 and sometimes up 40 mg/dL^11,12,14,23,32–35^.

Cholesterol can be taken up in cells via the LDL receptors and transported to intracellular membranes. In addition, mature erythrocytes can take up free cholesterol in their plasma membrane from the circulation in a dynamic fashion^36–38^. Mature RBCs plasma membranes are known to be a reservoir of cholesterol^39^.

In this study, we investigated the effect of cholesterol on in vitro erythropoiesis and on characteristics of cRBC with the ultimate goal to produce cRBC for transfusion purposes. Not only the concentration, but also the timing of cholesterol addition was important for a high yield of purified enucleated cRBC. Whereas early addition inhibited enucleation, the addition of up to 5 mg/dl cholesterol to culture medium at day 5 of differentiation, at the point of enucleation, enhanced osmotic stability and filtration efficiency of enucleated cRBC. We used Bodipy-labelled-Cholesterol to track the uptake and exchange of Cholesterol from the cRBC membrane by flow cytometry^50^.

## Materials and methods

### Cell culture

Human adult peripheral blood mononuclear cells (PBMCs) were isolated from donor derived buffy coats by density centrifugation using Ficoll-Paque (density = 1.077 g/mL; 600 g, 30 min; GE Healthcare). In accordance with the Declaration of Helsinki and the Sanquin Ethical Advisory Board this waste material was only used for research purposes when written informed consent was given by the donor, which was verified by the Sanquin Not For Transfusion (NVT) committee (approval file number NVT0258; 2012).

Erythroid cells were cultured from human PBMCs in serum-free medium as previously described^13^ with minor modifications to the Cellquin medium, which lacked nucleosides, and contained a defined lipid mix (Sigma-Aldrich Cat#L0288; USA 1:1000) replacing cholesterol, oleic acid, and L-a-phosphatidylcholine^15^.

In the first phase, (pro)erythroblast cultures were established from PBMC in the presence of erythropoietin (Epo, 1 U/mL; EPREX®, Janssen-Cilag, Netherlands), hSCF (30 ng/mL, produced in HEK293T cells), dexamethasone (1 µmol/L; Sigma-Aldrich), and IL-3 (1 ng/mL first day only; Stemcell Technologies; Canada) using 10 cm culture dishes. From day 6, erythroblast cultures were expanded and the cell density was maintained between 0.7 and 2.0 × 10^6^ cells/ml (CASY Model TCC; OLS OMNI Life Science; Germany).

To induce differentiation, cells were washed and reseeded at 1–2 × 10^6^ cells/ml in the presence of EPO (5 U/mL); 5% plasma (Omniplasma from Octapharma GmbH; Germany) and heparin (5 U/mL; Leo Pharma A/S; Denmark). During this differentiation phase the cells undergo 3–4 divisions before they mature to enucleated reticulocytes. Unless otherwise stated most experiments were performed on cells differentiated for 11 days with or without cholesterol in stated concentrations.

Cholesterol (Cholesterol Powder Bioreagent; Merck; Merck Millipore; stock 25 mg/ml in alcohol puro) was added as indicated. For absolute amounts of Cholesterol per culture condition see Table 1.

**Table 1:** Amount of cholesterol per culture condition.

Medium description	Cholesterol derived from chemically defined lipid mixture 1 (mg/dL)	Cholesterol derived from Omniplasma (mg/dL)	Extra cholesterol added (mg/dL)	Total cholesterol (mg/dL)
SEM	0.022			0.022
SEM + Cholesterol 2 mg/dL	0.022		2.0	2.0
SEM + Cholesterol 5 mg/dL	0.022		5.0	5.0
SDM	0.022	3.3		3.3
SDM + Cholesterol 2 mg/dL	0.022	3.3	2.0	5.3
SDM + Cholesterol 5 mg/dL	0.022	3.3	5.0	8.3

SEM: standard expansion medium. SDM: standard differentiation medium.

. .

**Figure 1:**
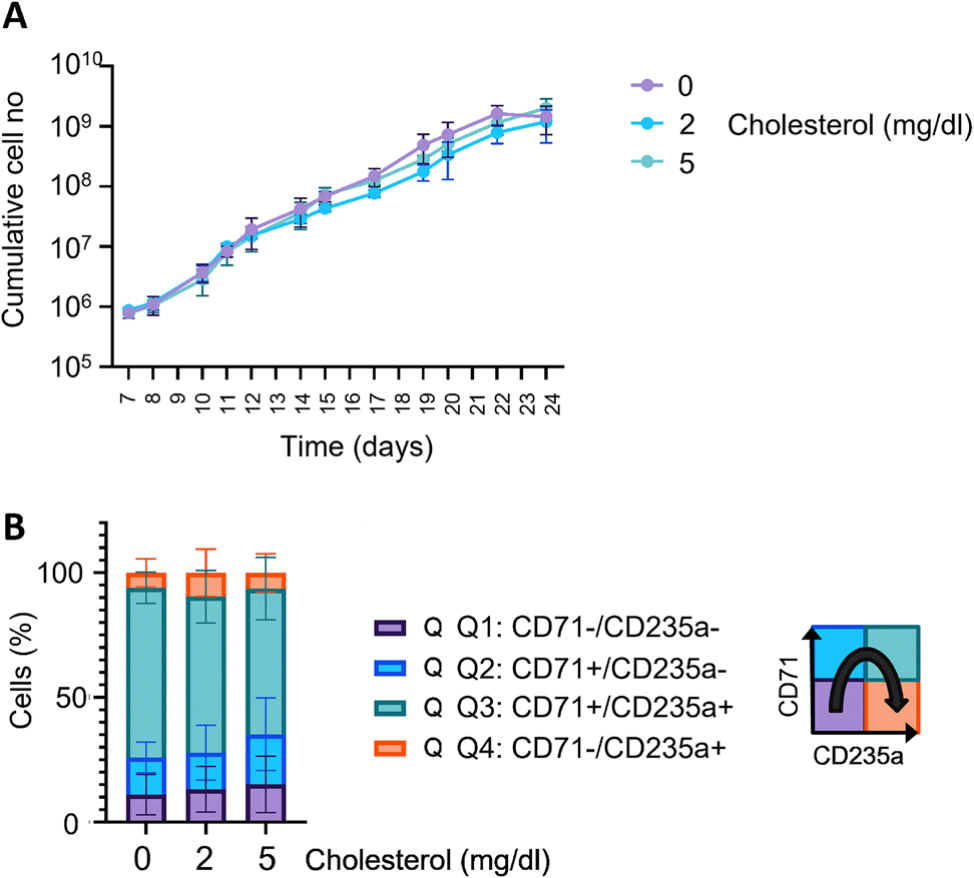
Cholesterol supplementation does not affect expansion of erythroblast cultures. Erythroblasts were cultured from PBMC in presence of Epo (1 U/ml), SCF and dexamethasone. Cholesterol was added on day 7 at 2.0 or 5.0 mg/dl. (**A**) Cells were counted at indicated time intervals and cell density was maintained between 0.5 × 10^6^/ml and 1.5 × 10^6^/ml through dilution with fresh medium (plus or minus cholesterol). Cumulative cell numbers were calculated (*n* = *3*). (**B**) Expression of transferrin receptor (CD71) and Glycophorin A (CD235a) in presence and absence of cholesterol was evaluated by flow cytometry on day 11. Percentage of cells in each quadrant is shown: Q1 (stage 1) CD71^neg^/CD235a^neg^ ; Q2 (stage 2) CD71^pos^/CD235a^neg^ ; Q3 (stage 3) CD71^pos^/CD235a^pos^ ; Q4 (stage 4) CD71^neg^/CD235a^pos^. Nine independent experiments; error bars indicate standard deviation.

### Bodipy-cholesterol uptake and wash-out experiments

To measure cholesterol uptake, erythroblasts cultured for 5 days in standard differentiation medium (SDM) were incubated for 2, 4, 8, 16 or 24 h in SDM medium containing Cholesterol 5 mg/dL and various concentrations of Bodipy-Cholesterol (TopFluor Cholesterol 23-(dipyrrometheneboron difluoride)-24-norcholesterol powder; Merck; Merck; Cat#810255P^41^) (0); 0.5; 2 or 5 µM. Bodipy-Cholesterol has similar properties to that of naïve cholesterol^40^. To measure cholesterol leakage, erythroblasts cultured for 5 days in standard differentiation medium (SDM) were incubated overnight in SDM without plasma containing Cholesterol 2 mg/dL and Bodipy-Cholesterol 2 µM. Cells were washed and resuspended in SDM without plasma containing Cholesterol 2 mg/dL (without Bodipy-cholesterol). Samples were taken at (0) 2, 4, 16 and 24 h.

### Flow cytometry

Cells were washed and resuspended in HEPES buffer + 0.5% BSA. Cells were incubated for 30 min at room temperature with antibodies directed against CD235a (PE-labeled, 1:2500 dilution; OriGene cat#DM066R), CD71 (APC labeled, 1:200 dilution; Miltenyi cat#130-d099-219; or VioBlu labeled, 1:1000; Milteny; Cat#130–128-947)); LDLR (APC labeled, (1:1000; Abcam; Abcam Cat#ab275614); or CD44-PE (1:50 Miltenyi Cat#130–113-335); CD49d-BV421 (1:100; BD Biosciences; Cat#565277). Alternatively, cells were stained with Hoechst (1:1000; Thermo Fisher; Invitrogen; Cat#H3570); DRAQ5 (nuclear stain; 1:2500 dilution; abcam cat#ab108410) or Bodipy-Cholesterol (Merck; Merck: Cat#810255P). Fluorescence was measured using a BD FACSCantoTM II flow cytometer or LSRFortessa (both BD biosciences, Oxford, United Kingdom), gated against specific isotypes, and analyzed using FlowJoTM (version 10.3; USA).

### Hemoglobin concentration

Hemoglobin (Hb) determination was based on its iron-dependent oxidation of o-Phenylenediamine in presence of hydrogen peroxide^42^. Absorbance of reaction product 2,3-diamiophenazine, at wavelength 425 nm was recorded. Arbitrary units were determined by Absorption/Cell count.

### Filter efficiency/recovery post filter

A leukocyte reduction filter (Acrodisc® WBC Syringe filter; VWR; Pall Laboratory, Cat#514–0682) was pre-wetted with 5 ml of Saline Adenine Glucose Mannitol (SAGM). The cultured cell suspension containing 50 million cells was loaded into the filter and collected (run through 1). Next the filter was loaded with 5 ml of SAGM and the run through was collected (run through 2). This last step was repeated once with 5 ml of SAGM (run through 3). Run through 1 and 2 contained the reticulocytes and were pooled and harvested via centrifugation on 600G for 5 min.

### Filter recovery calculations

The Recovery was calculated as a percentage of the total pre-filter cells: number of cells in run through 1 plus run through 2 (numerator) divided by the number of cells pre filter (50 million) (denominator) × 100%.

The Recovery from the nucleated fraction was calculated as a percentage of the enucleated cells prior to filtration: number of cells in run through 1 plus run through 2 multiplied by enucleation percentage (numerator) divided by the number of cells pre filter multiplied by the enucleation percentage (denominator).

### RBC deformability

Cultured RBC deformability was measured using an Automated Rheoscope and Cell Analyzer (ARCA), in which cells were elongated by shear flow in a viscous medium (Polyvinylpyrrolidone) between parallel glass plates^43,44^. Automated imaging algorithms were used to locate cells and to calculate cellular deformability. All experiments were performed at a shear stress of 3 Pa and 10 Pa, and for each experiment more than 3000 cells were analyzed and grouped in 30 bins according to increasing elongation or cell projection area (as a measure of membrane surface area). The extent of elongation (major cell radius divided by minor cell radius) was plotted against the normalized frequency of occurrence. As reference freshly harvested erythrocytes and reticulocytes were used. Reticulocytes are less deformable compared to erythrocytes, depending on their maturity assessed by CD71 expression and TO (Thiazole Orange) staining^13^. We employed CD71^low^/TO^pos^ reticulocytes as a reference value.

### Osmotic resistance/osmotic shock assay (hemolysis assay)

Cultured RBCs were incubated for 5 min at room temperature in various Sodium Chloride concentrations between 0 and 0.9%. Following centrifugation (5 min; 500G) the absorption of the supernatant measured at 412 and 690 nm and the delta was determined^45^. The value at 0.2% NaCl was set at 100%, and all other data expressed as a percentage thereof. The number of cells for this experiment was determined by the number of cells that gave an absorption of 0.7 when lysed in 0% NaCl (ca 1.75 million).

### Statistical analysis

Statistical analyses were performed using Analysis of Variance (ANOVA). Post hoc multiple comparison analysis were performed using Dunnett’s test to identify the pair with significant differences between the treatment group and the control group. All data in figures are displayed as mean ± the standard deviation of the measurements. The number of replicates is n ≥ 3 for all experiments, unless indicated differently. Significance is expressed as: ns for not significant differences, * for *p* < 0.05, ** for *p* < 0.01, *** for *p* < 0.001, **** for *p* < 0.0001. All measurements and the statistical analysis is added as supplemental data.

## Results

### Cholesterol supplementation is not required in the expansion phase of erythroblast cultures

Serum free media are generally supplemented with lipid fractions including cholesterol up to 7 and sometimes up to 40 mg/dL^23^. Our current (pro)erythroblast expansion medium contains 0.022 mg/dl cholesterol as part of a defined lipid mix. We supplemented this medium with (0), 2 and 5 mg/dl cholesterol to analyze the contribution of cholesterol supplementation to the production of cultured red blood cells (cRBC) (for timeline see Supplementary Fig. 1). First, we analyzed lineage commitment and expansion of erythroblast cultures from peripheral blood mononuclear cells (PBMC) in the presence of Epo, SCF and dexamethasone. (Pro)erythroblasts dominated the cultures from day 7 onwards. Cell cultures were diluted daily to a cell concentration of 0.7 × 10^6^/ml and cumulative cell numbers were calculated. Cholesterol supplementation did not alter the cumulative cell numbers over 24 days of culture (Fig. 1A). In the expansion phase, the number of CD71^high^ (transferrin receptor) cells increased exponentially. The cells gradually acquired CD235a at the cell surface (GPA, Glycophorin A) and developed from CD71^pos^/CD235^neg^ to CD71^pos^/CD235^pos^. A small fraction of cells entered spontaneous differentiation and gradually lost transferrin receptor expression (CD71^neg^/CD235^pos^) (Supplementary Fig. 2). Cholesterol supplementation did not significantly alter this process (Fig. 1B, Supplementary Fig. 2).

### Proliferation and hemoglobinization during erythroblast differentiation does not require cholesterol supplementation

**Figure 2:**
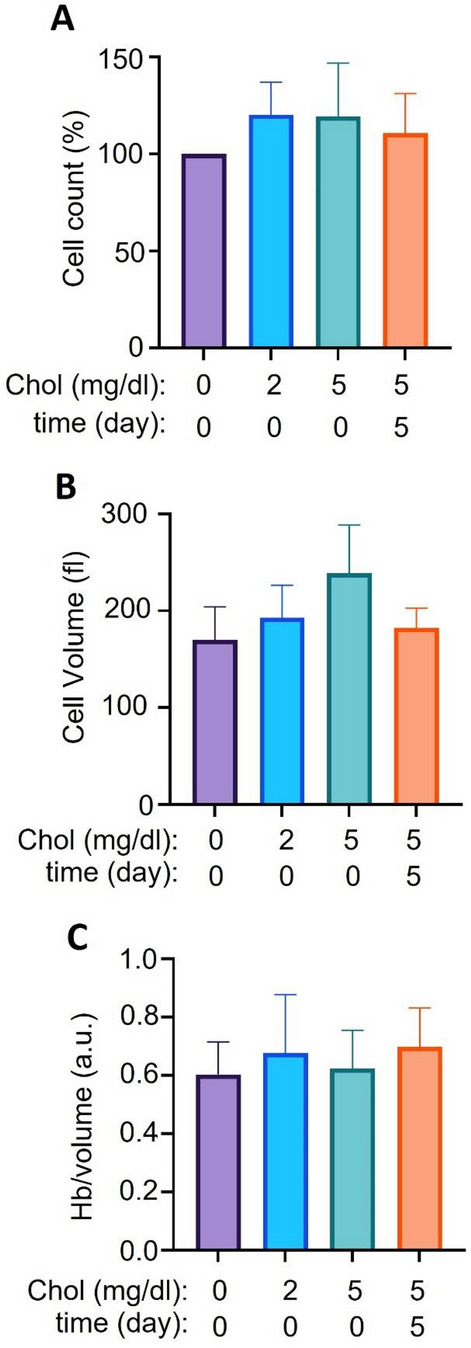
Cholesterol supplementation does not affect erythroblast differentiation. (**A**–**C**) Day 11 erythroblast cultures (Fig. 1B) were transferred to terminal differentiation conditions (plasma, heparin, Epo (5 U/ml)) for another 11 days with or without supplementation of additional cholesterol (2 or 5 mg/dl) at the start of differentiation (t = 0) or at day 5 of differentiation induction. Cell count (**A**) and cell volume (fl, femtoliter) (**B**) were measured on a CASY cell counter. The cell count of distinct cultures was normalized to the cell count of cultures without cholesterol supplementation. Hemoglobin was measured with a colorimetric reaction as arbitrary units (a.u.) and corrected for the cell volume (**C**). n = 3, Error bars indicate standard deviation, *p < 0.05.

Next, we induced terminal differentiation in the presence of increased EPO levels (5U/ml), increased holo-transferrin (1 mg/ml), and 5% plasma (Omniplasma). Cholesterol levels in plasma-containing medium were 3.34 mg/dL. We added (0), 2 or 5 mg/dL cholesterol to the culture medium resulting in concentrations of 5.34 and 8.34 mg/dL respectively at the onset of differentiation (for timeline see Supplementary Fig. 1). In addition, 5 mg/dL cholesterol was added on day 5 when enucleation was in progress^13^. We measured differentiation parameters at day 11 after induction of differentiation. Erythroblasts divide 2–4 times upon induction of terminal differentiation. This proliferation was not altered in presence of additional cholesterol (Fig. 2A). Of note, the mean volume of the cRBC increased with an increasing cholesterol concentration, but only when cholesterol was added at the start of differentiation induction (Fig. 2B). To analyze whether increased cell size was due to an inhibition of differentiation and possibly reduced enucleation or to the production of larger enucleated cRBC, the cell population was stained with the cell permeable DNA-dye DRAQ5, and enucleated cRBC were analyzed (Supplementary Fig. 3A). The forward scatter of DRAQ5 negative enucleated cells was increased in the presence of cholesterol 2 or 5 mg/dL added from the start of differentiation induction compared to no addition (+ 11% and + 12%, respectively) Supplementary Fig. 3B). The hemoglobin level per cell was increased (Supplementary Fig. 3C). This was due to the increase in cell size, because hemoglobin per cell volume was not altered (Fig. 2C).

### Cholesterol enhances the yield of enucleated cRBC

**Figure 3:**
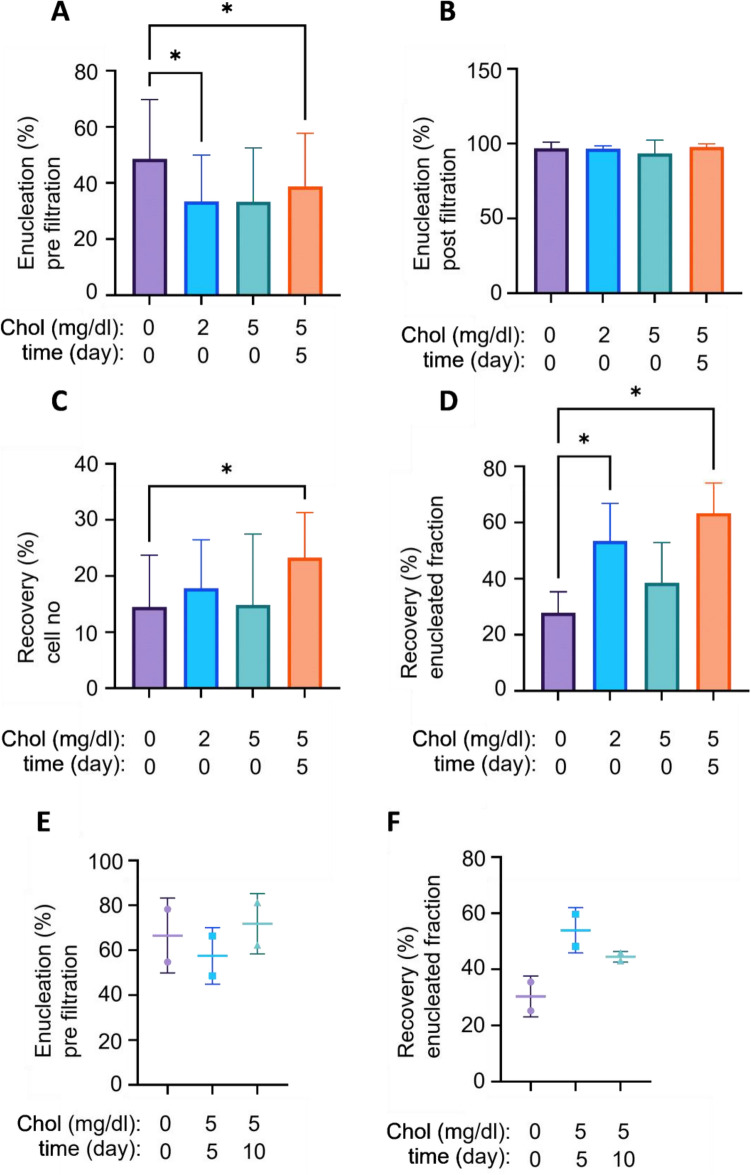
Cholesterol supplementation increases the yield of enucleated cRBC following filtration over a leukodepletion filter. Enucleated cells in cRBC cultures differentiated for 11 days in absence or presence of cholesterol (2 or 5 mg/dl; see Fig. 2) were analyzed by DRAQ5 staining and flow cytometry. (**A**, **B**) Enucleation efficiency was measured as the percentage of FSC-high DRAQ5 negative cells versus all FSC-high cells (supplemental Fig. 2A). Percentage enucleation pre-filtration (**A**) and post filtration (**B**). (**C**, **D**) The recovery of enucleated cRBC was calculated as percentage of the number of total cells prefiltration (C) and as percentage of the enucleated fraction prefiltration (**D**). n = 5, *p < 0.05, **p < 0.01. (**E**, **F**) Cholesterol (5 mg/dl) was added at day 5 and day 10 to differentiating erythroblast cultures. Enucleation (**E**) and Recovery from enucleated fraction (**F**) was measured similar to panels (**A**) and (**D**), respectively (n = 2).

The final stage of differentiation is enucleation of mature erythroblasts. Enucleated cRBC and extruded nuclei (pyrenocytes: tightly packed DNA surrounded by an erythroid plasma membrane) were separated on leukocyte depletion filters that are standardly used in transfusion technology to deplete leukocytes from erythrocyte transfusion units. We analyzed whether cholesterol supplementation could enhance the efficiency with which enucleated cRBC are recovered from filtration. Addition of cholesterol at 2 or 5 mg/dL at the initiation of erythroblast differentiation decreased enucleation efficiency (from 49 ± 21 to 34 ± 14% and 33 ± 17%, respectively). Addition of cholesterol at day 5 of differentiation, when enucleation was in progress, had a smaller but significant effect on enucleation (39 ± 19%) (Fig. 3A, Supplementary Fig. 4). The purity of enucleated cRBC post-filtration was similarly high under all conditions (Fig. 3B). Addition of cholesterol 5 days after differentiation initiation significantly increased the recovery of enucleated cRBC from the culture after filtration (from 14 ± 9 to 23 ± 8%, Fig. 3C). When recalculated to the initial number of enucleated cells cholesterol added either at day 0 or at day 5 both significantly increased the yield of enucleated cRBC post-filtration (from 28 ± 8 to 53 ± 13% in presence of 2 mg/dl cholesterol at day 0 and 63 ± 11% in presence of 5 mg/dl added at day5, Fig. 3D). This suggests that addition of cholesterol is most effective later in differentiation when enucleation is already in progress. Addition of cholesterol at day 10 following differentiation induction and filtered 24 h later, had no effect on total enucleation (Fig. 3E) but led to a similarly enhanced filtration efficiency (Fig. 3F).

### Deformability and stability of cRBC are enhanced by cholesterol

**Figure 4:**
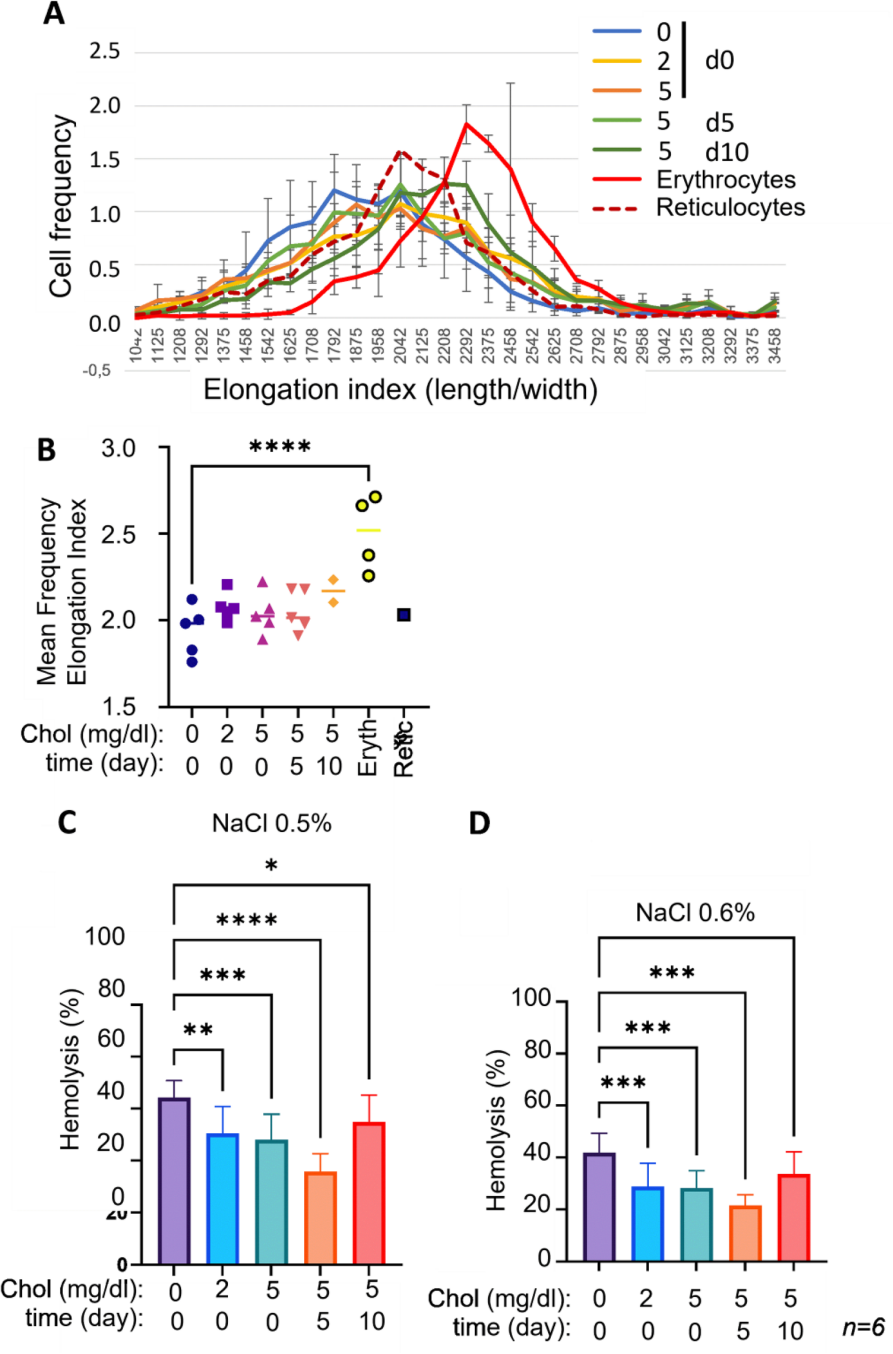
Cholesterol supplementation increases deformability and isotonic stability of cRBC. (**A**–**C**) Enucleated cells in cRBC cultures differentiated for 11 days in absence or presence of cholesterol (2 or 5 mg/dl, added at the start (d0) or at day 5 or day 10 after induction of differentiation; see Fig. 2) were purified. (**A**, **B**) Analysis by automated rheoscope and cell analyzer (ARCA) at a shear stress of 3 Pa. The elongation under shear stress was compared to freshly isolated erythrocytes and reticulocytes from peripheral blood. (**C**, **D**) Hemolysis assay at NaCl concentrations of 0.5% w/v (**B**) and 0.6% w/v (**C**). n = 6, error bars indicate standard deviation.

Increased recovery of enucleated cRBC during filtration may indicate altered membrane properties and possibly enhanced flexibility and stability of cRBC. Deformability of enucleated cRBC (post-filtration) was measured using an ARCA (Automated Rheoscope and Cell Analyzer), which measures the length over width ratio of individual cells under shear stress as a measurement of shear-induced deformability. Medium supplementation with cholesterol shows a trend improved the deformability of enucleated cRBC at all concentrations (Fig. 4A, B). Addition of cholesterol at day 10 of differentiation, 24 h before harvest, resulted in the most deformable cRBC population although not statistically significant compared to SDM. This cRBC population was more deformable than the reticulocyte reference sample. Because deformability and stability are associated, we also tested the osmotic resistance of post-filtration enucleated cRBC in comparison to freshly isolated erythrocytes. Hemolysis decreased with increasing NaCl concentration (Supplementary Fig. 5). The differences in hemolysis between the various samples were most pronounced at 0.5 and 0.6% NaCl (Fig. 4C, D). Addition of cholesterol increased osmotic resistance when cholesterol was supplemented to the medium on either day 0 or at day 5 of differentiation. However, addition of cholesterol 24 h prior to harvesting (day 10) did not increase osmotic resistance in comparison to control cRBC (Fig. 4B, C).

### Uptake of cholesterol from the medium

**Figure 5:**
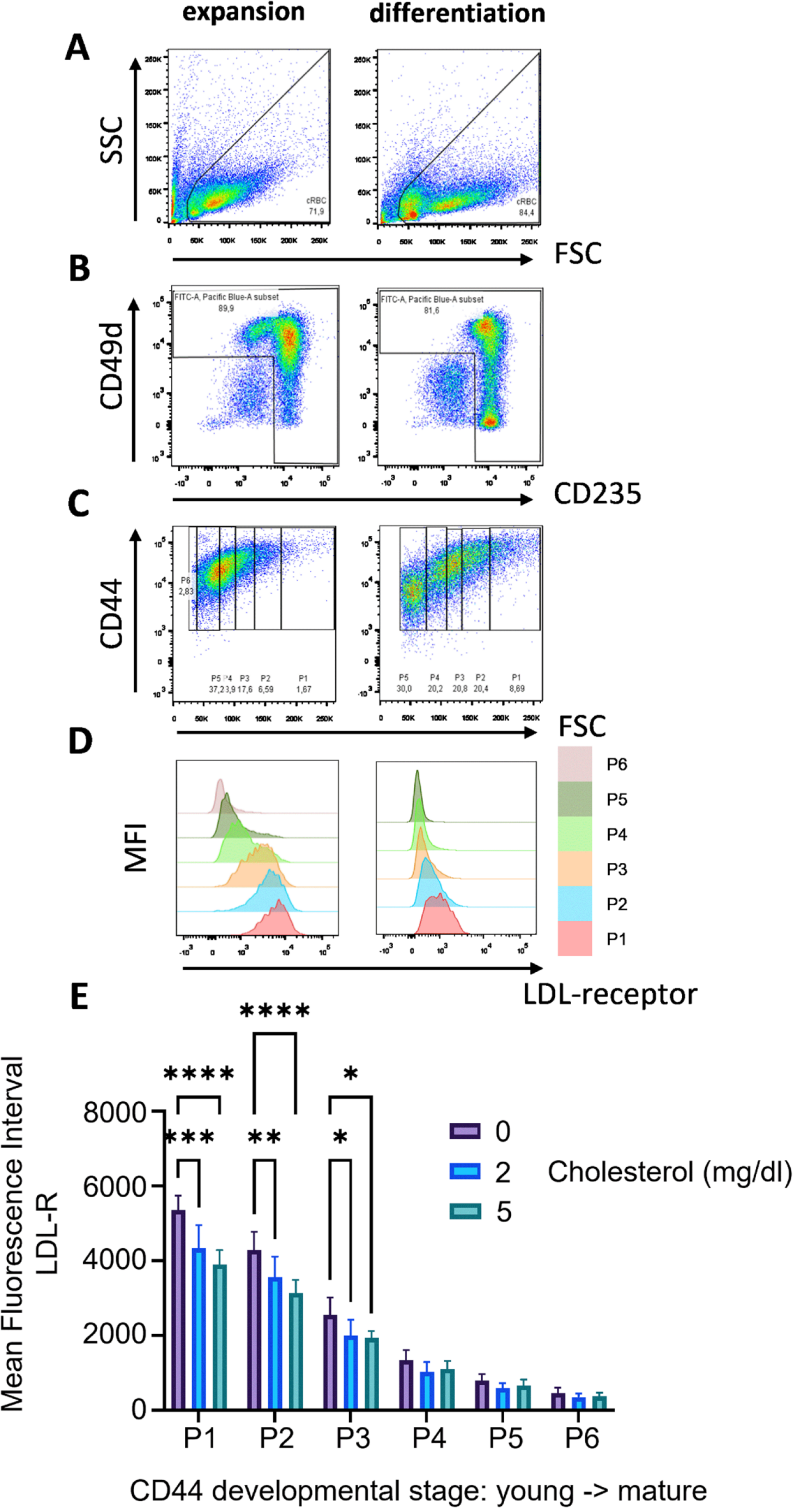
LDL-receptor expression during erythroblast differentiation. Erythroblast cultures in expansion medium (day 11 from seeding PBMC; left panel) and erythroblasts subsequently differentiated for 3 days (right panel) were analyzed by flow cytometry for LDL-receptor expression. (**A**–**C**) Gating strategy: (**A**) selected live cells based on forward scatter (FSC) versus side scatter (SSC). (**B**) Within live cells the selection of cells expressing Glycophorin A (CD235a) or integrin subunit α4 (CD49d). (**C**) On this erythroblast selection FSC versus CD44 were visualized and divided in 6 subpopulations (P1 to P6) with increasing maturity (CD44 decreases from proerythroblast (P1) to reticulocyte (P6). (**D**) Histogram overlay of Mean Fluorescence Intensity (MFI) of LDL Receptor during developmental stages (P1 through P6). (**E**) MFI of LDL-R expression in P1–P6 as in (D) for cultures without and with supplementation of cholesterol (2 and 5 mg/dl) Error bars indicate standard deviation (n = 3).

Cholesterol is mostly present in lipoprotein complexes (LDL, VLDL) and internalized via the LDL receptor. Therefore, we measured LDL-receptor expression in cell populations cultured under expansion conditions (day 11) and upon induction of differentiation (day 3). Cells were stained for expression of LDL-receptor plus CD49d/integrin alpha4, CD235/GPA and CD44, and analyzed by flow cytometry (Fig. 5). Distinct subpopulations were selected first on cell size (Fig. 5A), then on expression of CD49d/integrin alpha4 or CD235/GPA (Fig. 5B), and subsequently on CD44 expression and size (FSC), which represented increasingly more mature populations as CD44 and FSC decreased (Fig. 5C)^46^. Increased differentiation was associated with decreasing expression of the LDL-receptor (Fig. 5D). Next, the expression of the LDL-receptor was assessed in cells cultured with or without cholesterol supplementation. In populations with decreasing CD44 expression, and decreasing size, the LDL-receptor surface expression was reduced to negligible levels (Fig. 5E). Interestingly, the lower expression of the LDL-receptor in presence of cholesterol suggests cholesterol-induced internalization of the LDL-receptor, and thus LDL-receptor-mediated uptake of cholesterol, particularly at earlier stages of differentiation (Fig. 5E).

**Figure 6:**
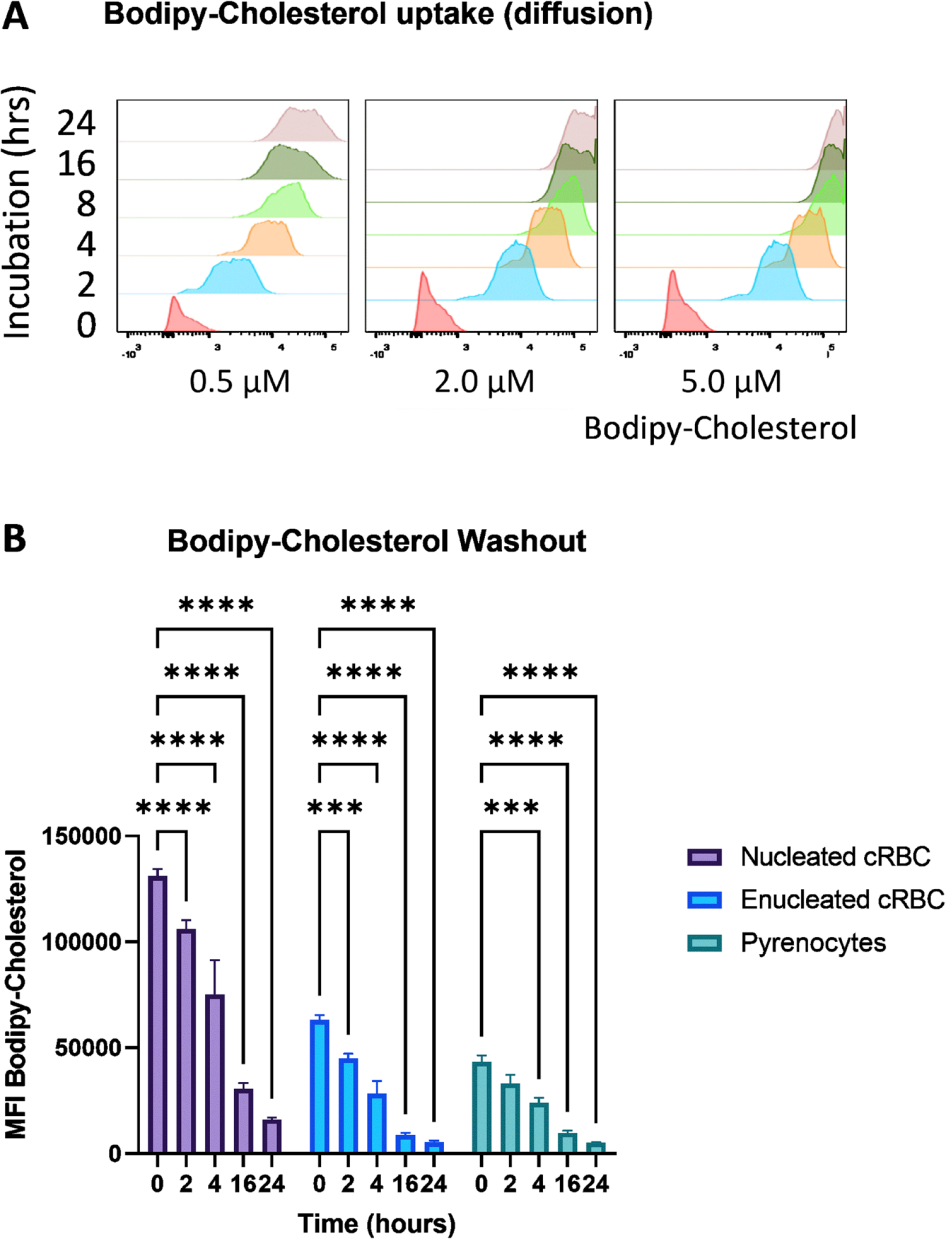
Rapid time and concentration dependent uptake of cholesterol in differentiating cRBC cultures. Cultures were exposed to differentiation conditions in presence of 5 mg/dl cholesterol for 5 days. Bodipy-labeled cholesterol was added at 0.5, 2.0 and 5.0 µM for increasing incubation times 0, 2, 4, 8, 16, and 24 h). (**B**) 5 days differentiated cRBC cultures were incubated overnight in presence of Cholesterol 2 mg/dL plus Bodipy-Cholesterol 2 µM. After overnight incubation, cells were washed and resuspended in medium containing Cholesterol 2 mg/dL (without Bodipy). Bodipy-Cholesterol Wash-Out Samples were taken at 0, 2, 4, 16, 24 h. Enucleated cRBC, Nucleated cRBC, Pyrenocytes distinguished on the basis of DRAQ5 and Forward Scatter.

Strikingly, cholesterol supplementation improved the yield of enucleated cRBC when it was added at day 5 of differentiation when LDL-receptor expression was very low to absent. Therefore, diffusion of free cholesterol directly into the cRBC membrane may also occur. Trace amounts of bodipy-labeled cholesterol, together with unlabeled cholesterol 5 mg/dl, were supplemented to cultures 5 days after differentiation induction. Rapid and concentration dependent uptake of bodipy-cholesterol was detected by flow cytometry (Fig. 6A) (and Supplementary Fig. 6A).

Next, cells that were differentiated for 3 days were subsequently incubated overnight in differentiation medium containing bodipy-cholesterol 2 µM and cholesterol 2 mg/dL. Flowcytometry showed uptake in both nucleated and enucleated cRBC (Supplementary Fig. 6B, C) and pyrenocytes (Supplementary Fig. 6B, C). Subsequently cells were transferred to medium without Omniplasma containing only cholesterol 2 mg/dL (no bodipy-cholesterol). The bodipy-cholesterol disappeared rapidly from the cell membrane (Fig. 6B, Supplementary Fig. 6D). The loss of bodipy-cholesterol was slightly faster in enucleated cells, with significantly more loss in enucleated cells at the later timepoints. Given the large overall loss, however, this does not seem biologically relevant (Supplementary Fig. 6E).

## Discussion

The purification of enucleated cells is a crucial processing step in the production of cRBC. Separation of pyrenocytes (the extruded nuclei) and enucleated cells using leukocyte depletion filters resulted in a large loss of enucleated cRBC. Supplementing the culture medium with cholesterol increased the recovery rate of enucleated cRBC from leukodepletion filters, which was associated with increased deformability and osmotic stability. It was important to add cholesterol at day 5 following induction of differentiation was optimal for this process because earlier addition inhibited enucleation. Cholesterol may directly diffuse into the cRBC membrane because the LDL-receptor is downregulated during terminal erythroid differentiation. Addition of trace amounts of bodipy-cholesterol showed a time and concentration dependent, rapid uptake and release of the bodipy-cholesterol from cRBC.

We observe some variation between donors and between experiments in the enucleation kinetics, which is subsequently reflected in the yield of enucleated cRBC after filtration at day 11 as shown in this manuscript. We previously showed enucleation rates of up to 90%^13^. Lower enucleation rates at day 11 mostly indicate slower kinetics, and an enucleation rate that still increases on day 12 or 13 of differentiation. However, we maintained conditions constant for all donors and in all experiments. The addition of cholesterol increased the filtration efficiency irrespective of enucleation kinetics. Eventually, the production of cRBC for transfusion will make use of stirred-tank bioreactors, which showed more reproducible results^15^. Application of shear also resulted in efficient and reproducible enucleation^47^.

During the expansion phase of erythroblast cultures, we use dexamethasone which may have a negative influence on lipid biosynthesis^48,49^. This may render synthesis of the plasma membrane of erythroid cells dependent on lipid-uptake from external sources^49^. However, we did not observe a significantly increased and prolonged expansion capacity in response to the addition of cholesterol that was previously described^12,49^. Erythroid progenitors are able to synthesize cholesterol, whereas they are also able to take up cholesterol-rich particles through the LDL receptor. During the expansion phase, both mechanisms may be regulated by culture conditions that we do not yet understand and control in detail.

The addition of LDL, which is taken up by the LDL-R, abrogated the requirement of plasma or serum at early stages of terminal differentiation^50^. All our differentiation experiments are performed in the presence of 5% plasma that is critically required for enucleation in our cultures. Therefore, we do not detect an effect of cholesterol on cell number during the first days of terminal differentiation. Wang et al. suggest that supplementation of cholesterol is not required during late terminal differentiation. We specifically increased the cholesterol concentration in our media at late stages for more efficient recovery of enucleated cells after filtration, which is not tested in their report^50^.

The amount of cholesterol used in our differentiation conditions ranged between 0.022 and 8.3 mg/dL. Bernecker et al., showed that supplementation of cholesterol up to 7 mg/dL resulted in a near to normal cholesterol content in the cRBC compared to naïve red blood cells^23^. The addition of Cholesterol in the culture medium is of importance in the downstream processing of the cRBC and is likely to affect the in vitro stability during storage. Our data suggests free exchange of cholesterol between medium and the cell membrane of differentiated cRBC that lack the LDL-receptor. This may be facilitated by liposomes created by the solubilization of lipids in water and that are absorbed by cultured cells through diffusion^14^. This is in accordance with previous observations in which erythrocyte cholesterol content reflects plasma cholesterol levels and regulates oxygen diffusion and ion transport through the erythrocyte membrane^51–53^. Cholesterol accumulates in specific microdomains of the erythroid membrane, preferentially in high curvature areas of erythrocytes, and controls reshaping of erythrocytes under flow^54^. Interestingly, hypercholesteremia is associated with stiff membranes whereas we and others observed an increased deformability in the presence of cholesterol, which indicates that there must be an optimum membrane cholesterol content to enable erythrocyte deformability. Culture medium, however, contains less cholesterol compared to 100% blood plasma. Following transfusion, cRBC may probably take up cholesterol and be part of the reverse cholesterol transport from tissues to blood because serum cholesterol level are at average 15 times higher compared to our culture medium^55,56^. This also implies that addition of cholesterol does not hold any risk of increasing the cholesterol load of the patients.

To purify the culture product and separate the enucleated cRBC from the nucleated cRBC, pyrenocytes and debris, we used a standard leukodepletion filter as used by many blood banks in the standard processing of blood donations. Standard filtration technology results in a loss in RBC number of up to 10 to12%^57^. In experiments with cRBC we only used a fraction of a standard blood donation. The loss due to non-specific adhesion of RBC may be similar in absolute numbers and therefore much larger as a percentage of a small product^58^. The addition of cholesterol to the culture medium did not affect the purity after filtering but significantly increased the yield of enucleated cRBC comparable to what was shown by Bernecker et al^23^. Going forward, there are also other innovations to purify the culture such as a tangential flow filtration system, an acoustic resonance cell filtration or a lab-on-a-chip microfluidic label free reticulocyte sorting method, in which the requirements for cRBC stability and flexibility may differ^59–62^.

Ultimately, the stability of our transfused cRBCs will be tested in a clinical phase I trial which is in preparation. In this phase-I clinical feasibility study we will test in parallel the 24 h survival of biotinylated cRBCs to biotinylated fresh nRBC of the same volunteer.

Conclusion: The addition of cholesterol to our SEM and SDM results in an increased yield of cRBC due to reduced loss during the purification process by leukodepletion filters. It also resulted in more deformable and osmotic stable cRBC. Although the addition of cholesterol increases the cRBC cell size the hemoglobin level per cell volume remained constant.

### Supplementary Information

Supplementary Information 1.

Supplementary Information 2.

## Data Availability

For original data, please contact m.vonlindern@sanquin.nl. FAIR website Sanquin.

## Abbreviations

APC-A: Allophycocyanin
ARCA: Automated rheoscope and cell analyzer
ATP: Adenosine triphosphate
PBMC: Peripheral blood mononuclear cell
CD235a: Glycophorin A
CD44: Indian blood group system, transmembrane protein, adhesion molecule
CD49d: Integrin alpha 4
CD71: Transferrin receptor 1
cRBC: Cultured red blood cell
Dex: Dexamethasone
DRAQ5: 1,5-Bis{[2-(di-methylamino)ethyl]amino}-4, 8-dihydroxyanthracene-9,10-dione, a cell permeable far-red fluorescent DNA dye used in live or fixed cells
EPO: Erythropoietin
FC: Fold change
FITC-A: Fluorescein isothiocyanate
FSC: Forward scatter
GPA: Glycophorin A
Hb: Hemoglobin
HDL: High-density lipoprotein
hSCF: Human stem cell factor
LDL: Low-density lipoprotein
NaCl: Sodium chloride
NVT: Not for transfusion
PBS: Phosphate-buffered saline
RBC: Red blood cell
Rh: Rhesus
Rpm: Revolutions per minute
SAGM: Saline adenine glucose mannitol
SCF: Stem cell factor
SDM: Standard differentiation medium
SEM: Standard expansion medium
SR-BI: Scavenger receptor class B type I
TO: Thiazole orange
VLDL: Very low-density lipoprotein
