# Supplementary material for: Production and stability of cultured red blood cells depends on the concentration of cholesterol in culture medium
Source: Sci Rep. 2024 Jul 6;14:15592. doi: 10.1038/s41598-024-66440-z (PMC11227516; doi:10.1038/s41598-024-66440-z)
Supplement: Supplementary file 2 — Supplementary Information 2. [file 41598_2024_66440_MOESM2_ESM.pdf]

Supplementary Fig. 1

Schematic overview cRBC culture system and timeline of additions and experiments

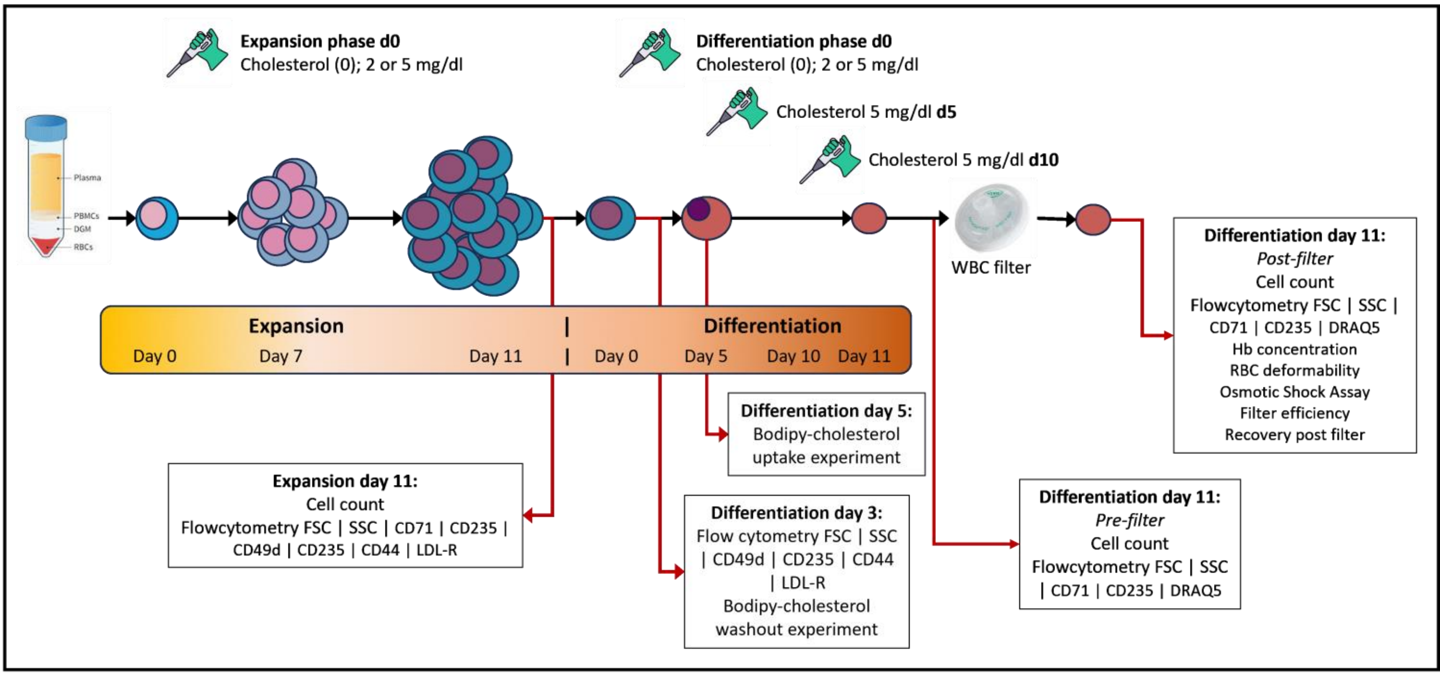

Schematic overview of cRBC culture system timeline of cholesterol additions and additional experiments.

Supplementary Fig. 1:

Schematic overview of red blood cell culture in expansion phase and subsequently differentiation phase. The addition of Cholesterol (0), 2 or 5 mg from day 0 to the expansion medium; or Cholesterol (0), 2 or 5 mg from day 0; or Cholesterol 5 mg from day 5 or 10 to the differentiation medium. The purification (using leukoreduction filter) after harvest at day 11 of differentiation. And the timepoints of additional testing (expansion day 11, differentiation day 3, differentiation day 5, differentiation day 11 pre- and post-filter).

Supplementary Fig. 2

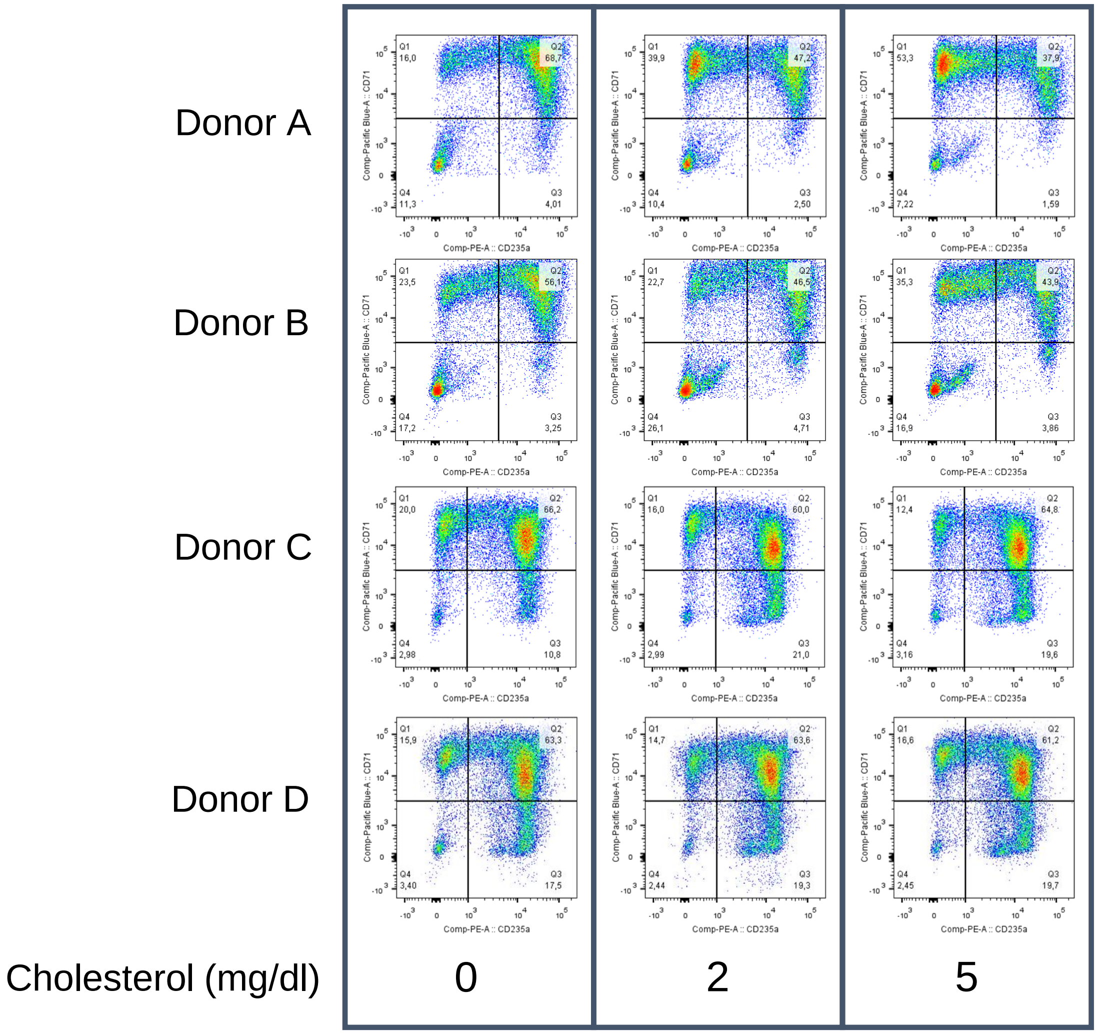

Supplementary Fig. 2:

Cultured Red Blood Cells in Standard Expansion Medium (SEM) or SEM with added cholesterol 2 or 5 mg/dL. Flow Cytometry populations at start differentiation (day 11 expansion) of 4 different donors (A, B, C, D).

Supplementary Fig. 3

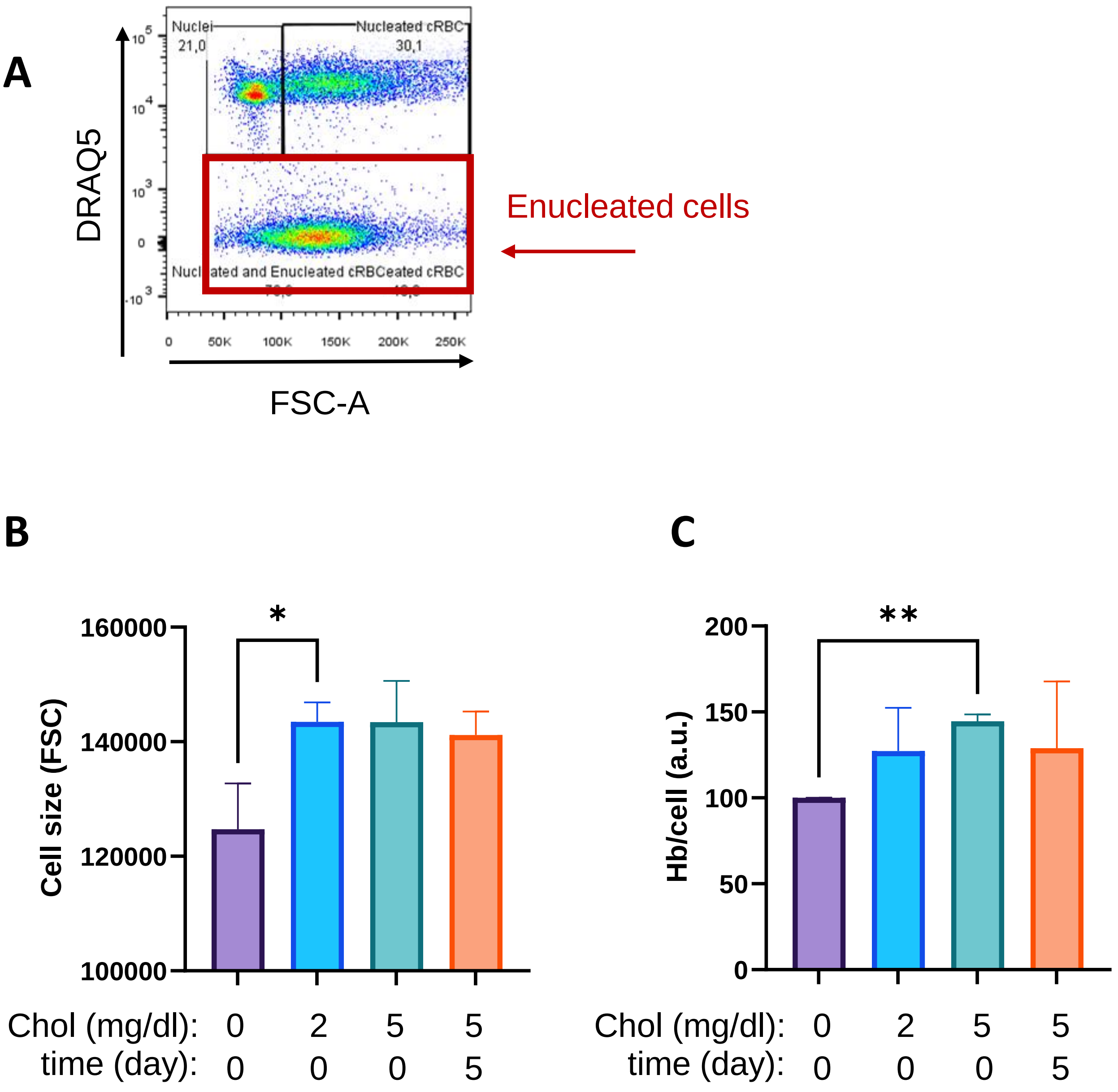

Supplementary Fig. 3:

Cultured Red Blood Cells differentiated (day 11) in standard differentiation medium (SDM) or SDM with added cholesterol 2 or 5 mg/dL from start differentiation (day 0) or Cholesterol 5 mg/dL from day 5 of differentiation.

Flow Cytometry Forward scatter versus DRAQ5 (nucleic stain) (A). Gating strategy: live cells, Transferrin Receptor and/or Glycophorin positive cells (CD71+ and/or CD235a+). Cell size (forward scatter) (B) and Hemoglobin per cell (C). Error bars indicate standard deviation.

Supplementary Fig. 4

A

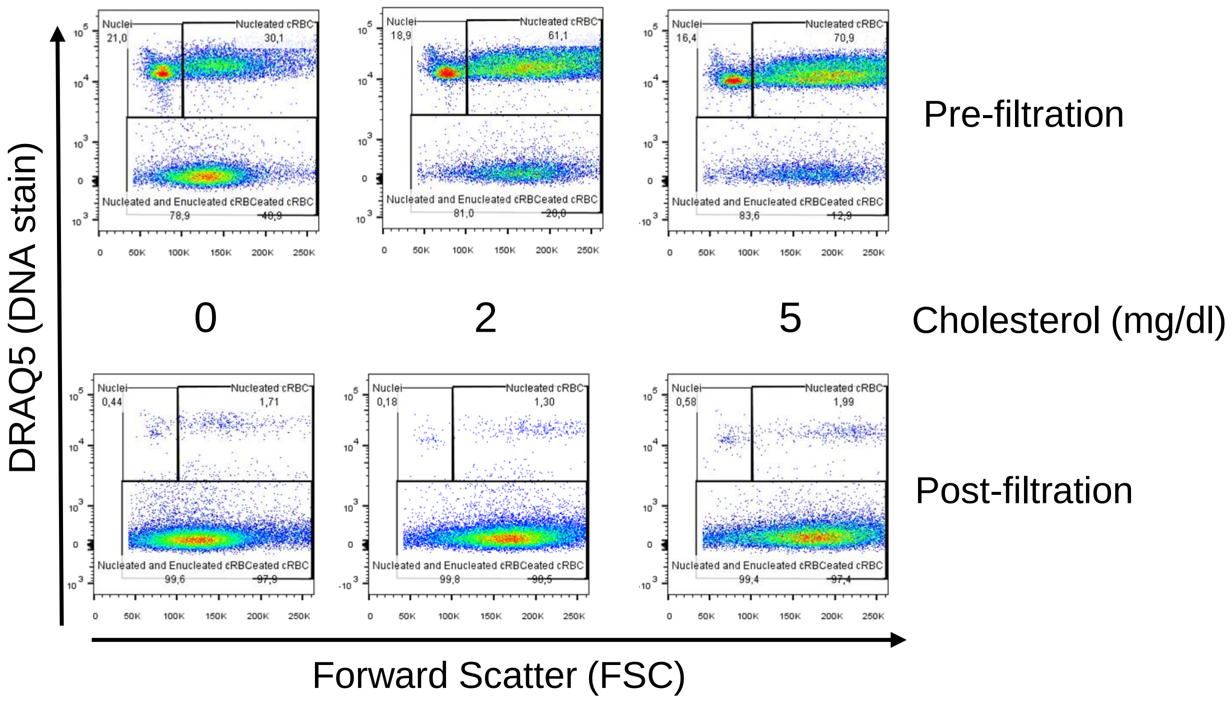

Supplementary Fig. 4:

Cultured Red Blood Cells differentiated (day 11) in standard differentiation medium (SDM) or SDM with added cholesterol 2 or 5 mg/dL from start differentiation (day 0).

Flowcytometry Forward Scatter (FSC) versus DRAQ5 (DNA stain) pre-filtration (top) and post-filtration (bottom) (A). Gating strategy: Live cells, Transferrin and/or Glycophorin A positive cells (CD71+ and/or CD235a+).

Supplementary Fig. 5

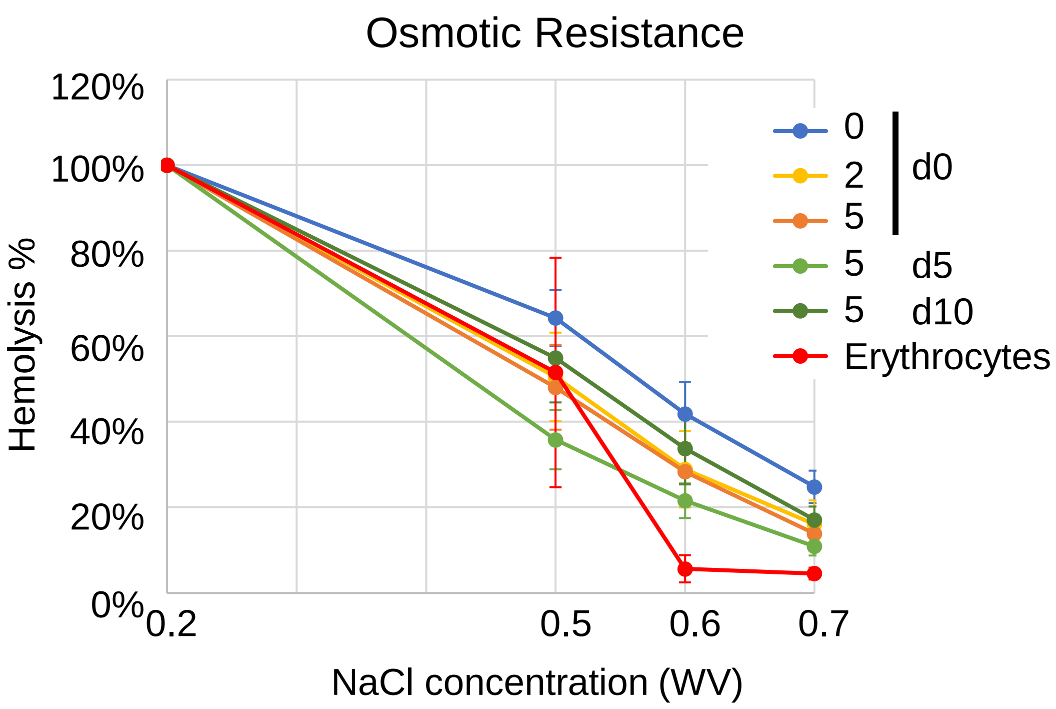

Supplementary Fig. 5:

Hemolysis assay of cultured Red Blood Cells differentiated (day 11) in standard differentiation medium (SDM) or SDM with added cholesterol 2 or 5 mg/dL from start differentiation (day 0) or Cholesterol 5 mg/dL from day 5 or Cholesterol 5 mg/dL from day 10 of differentiation; hemolysis is given at NaCl 0.5 – 0.6 and 0.7%. Error bars indicate standard deviation.

Supplementary Fig. 6

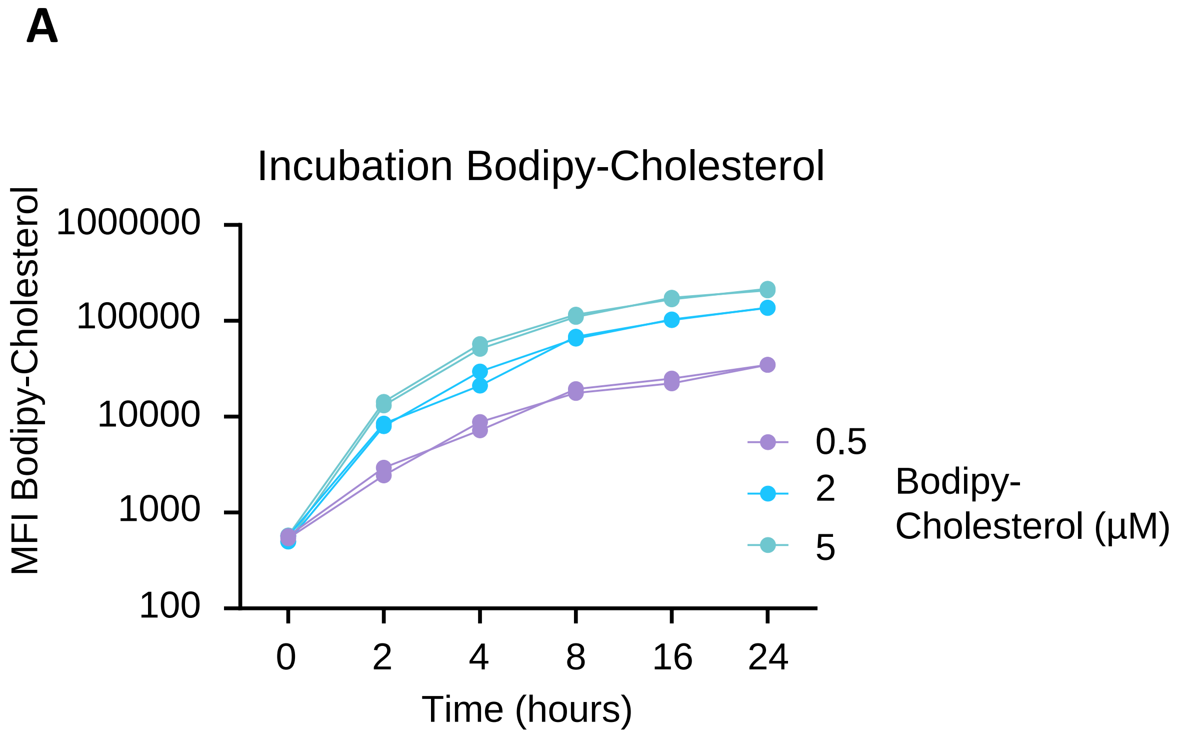

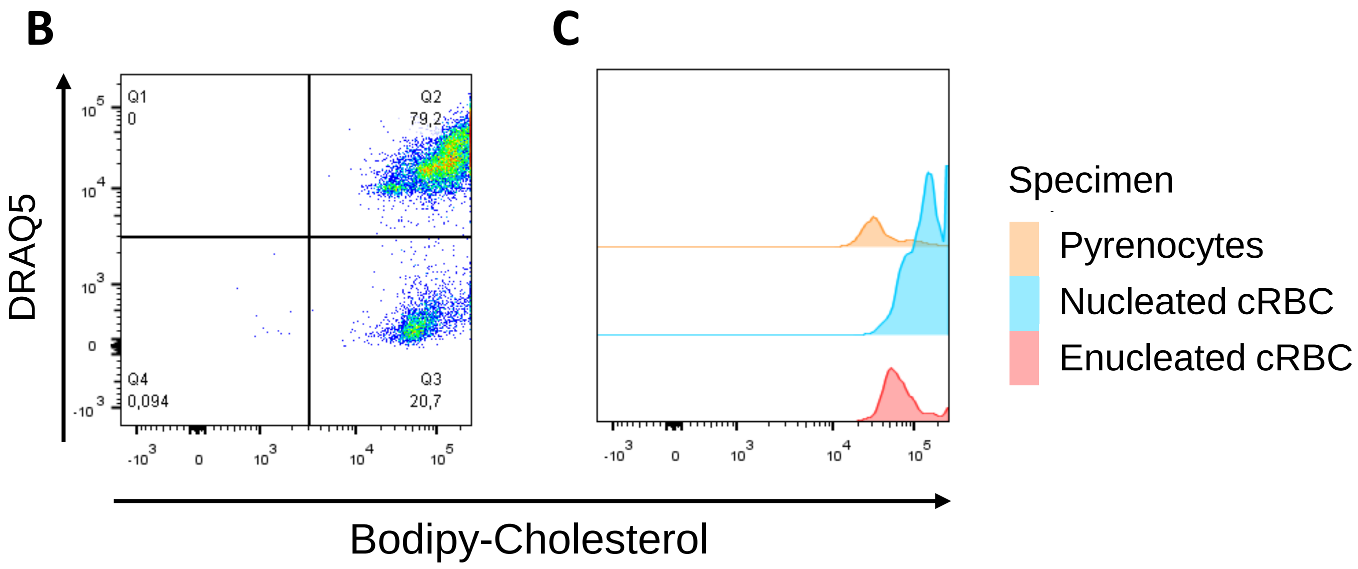

Supplementary Fig. 6

D

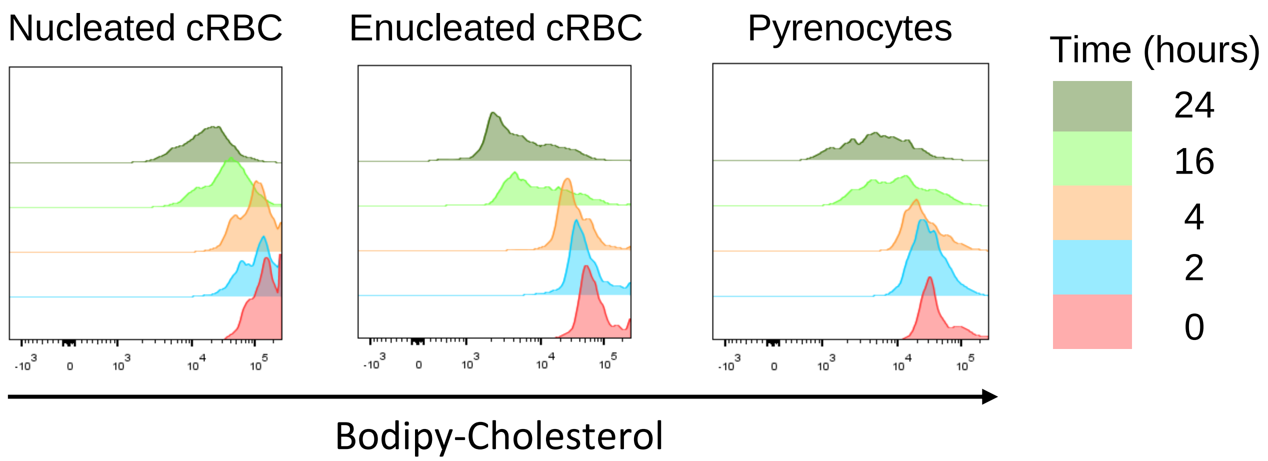

E

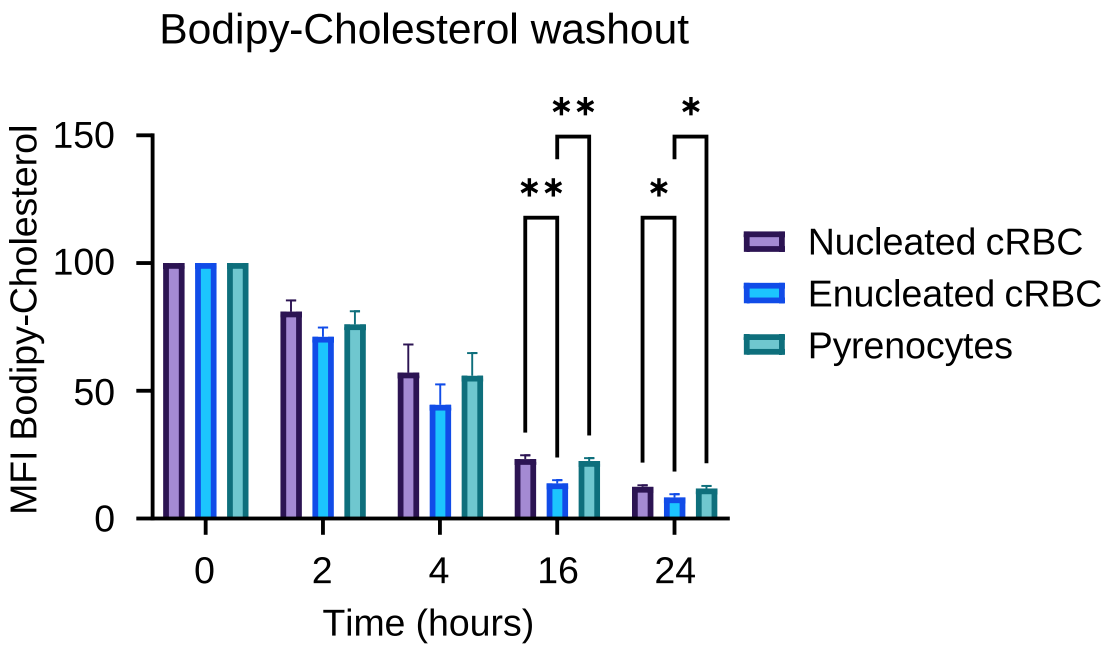

Supplementary Fig. 6:

cRBC incubated with different concentrations Bodipy-Cholesterol. Line diagram of time versus MFI Bodipy-Cholesterol in 2 donors (A). Bodipy-Cholesterol (FITC-A) versus nucleic stain DRAQ5 dot plot (S5B) and histogram overlay (S5C). Time dependent histogram overlay of Bodipy-Cholesterol in different populations of Enucleated cRBC, Nucleated cRBC, Pyrenocytes (D). Velocity of Bodipy-Cholesterol wash-out in the different populations (Nucleated cRBC / Enucleated cRBC / Pyrenocytes) (E).
